# Long-term use of rituximab increases T cell count in MS patients

**DOI:** 10.3389/fimmu.2024.1412668

**Published:** 2024-07-17

**Authors:** Gunnar Sigfús Björnsson, Hildur Sigurgrímsdóttir, Sólrún Melkorka Maggadóttir, Berglind Ósk Einarsdóttir, Ólafur Árni Sveinsson, Haukur Hjaltason, Sigurveig Þóra Sigurðardóttir, Björn Rúnar Lúðvíksson, Siggeir Fannar Brynjólfsson

**Affiliations:** ^1^ Faculty of Medicine, University of Iceland, Reykjavik, Iceland; ^2^ Department of Immunology, Landspítali – The National University Hospital of Iceland, Reykjavik, Iceland; ^3^ Department of Neurology, Landspítali – The National University Hospital of Iceland, Reykjavik, Iceland

**Keywords:** rituximab, B cells, T cells, CD4, CD8, multiple sclerosis (MS)

## Abstract

Rituximab has been used to treat MS patients in Iceland for over a decade. However, long-term effect of rituximab on leukocyte populations has not yet been elucidated. By retrospective analysis of flow cytometric data from 349 patients visiting the neurological ward at The National University Hospital of Iceland from 2012 to 2023 for rituximab treatment, the long-term effect of rituximab and whether the effect was dose dependent (1000mg vs 500mg) was evaluated. No difference was detected in efficacy of B cell depletion in patients treated with 500mg as an initial dose of rituximab when compared to 1000mg. Long-term use of rituximab led to an increase in T cell count (p=0,0015) in patients receiving 3-8 doses of rituximab (1.5-8 years of treatment). The increase occurred in both CD4^+^ (p=0,0028) and CD8^+^ T cells (p=0,0015) and led to a decrease in the CD4/CD8 ratio (p=0,004). The most notable difference lies in reshaping the balance between näive and effector CD8^+^ T cells. The clinical implications of long-term treatment with rituximab and its effect on the T cell pool needs to be explored further. Since no difference in B cell depletion was detected between the two patient groups, 1000mg as an initial dose might be excessive, suggesting a personalized dosing regimen might have therapeutic and financial advantages.

## Background

In Iceland, the monoclonal, chimeric anti-CD20 IgG1 antibody rituximab (RTX), has been used as an off-label treatment for multiple-sclerosis (MS) during the last decade and has increased considerably during that time. RTX has been shown to be superior to certain other disease-modifying treatments against MS (1), where a dose of 500-1000mg every 6-12 months was effective in the depletion of B cells and slowing down disease activity (2). RTX has shown benefits for MS patients in a phase 3 clinical trial, with no safety concerns (3). The immediate and long-term impact that such confound B cell depletion has upon safety, treatment efficacy, lymphocyte subsets re-population, and secondary lymphoid homeostasis is not fully known (4, 5). The aim of this study was to investigate the long-term and potentially dose dependent impact of RTX treatment on leukocyte populations in MS patients.

## Methods

### Cohort

In this retrospective study we analyzed flow cytometry data from 349 patients visiting the neurological ward at Landspitali – The National University Hospital of Iceland during the year 2012 until June 2023 (**Table 1**). Differential count data was analyzed for 286 patients receiving rituximab, including 262 patients diagnosed with MS and 24 diagnosed with other neurological diseases (**Table 1**). Two patients were excluded from the study, one due to a chronic lymphocytic leukemia (CLL) diagnosis and one due to a 300mg dose of RTX. The study was approved by the Landspítali – The National University Hospital Scientific Research Committee (42/2023).

**Table 1:** Patient demographics.

Patient demographics	(n=349)
Male, n (%)	109 (31)
Female, n (%)	240 (69)
Patients with differential count results after drug administration	286 (82)
Male, n (%)	95 (33)
Male age range (y)	17-76
Males diagnosed with MS, n (%)	82 (86)
Males, other neurological diseases, n (%)	13 (14)
Female, n (%)	191 (67)
Female age range (y)	16-84
Females diagnosed with MS, n (%)	180 (94)
Females, other neurological diseases, n (%)	11 (6)
Patient excluded from cohort due to CLL	1
Patients, 1000mg RTX for the first administration	133 (47)
Male, n (%)	49 (37)
Female, n (%)	84 (63)
Patients, 500mg RTX for the first administration	152 (53)
Male, n (%)	45 (30)
Female, n (%)	107 (70)
Patients, 300mg RTX for the first administration	1, male (1)

Data was collected from 349 patients. 286 patients had results from leukocyte differential count after RTX administration and were therefore eligible for the study.

The clinical decision on treating MS patients with rituximab is based on age, symptoms, radiological activity, type and duration of MS, and previous treatment(s). Rituximab has been used in Iceland for just over a decade for patients with moderate/high MS disease activity, those who have had disease breakthrough on other treatments, tolerability issues or JC virus positivity. Except for the MS diagnosis no other clinical data was retrieved. Rituximab is the most used disease modifying treatment for MS in Iceland and very efficacious. According to two of the authors experience (HH, ÓÁS who are experienced MS clinicians), it rarely happens that patients have an MS-relapse or new MRI lesions during treatment. To give a clinical picture of our study group we estimate that <5–10% of patients are graded 6 or higher on EDSS (disability scale widely used in MS research, 6=use a walking aid). The rituximab treatment followed a scheme from the Karolinska hospital in Stockholm. Initially we treat every 6 months for at least 2-3 times (using 500 or 1000 mg, mostly depending on disease activity). The aim is to keep CD19 cells depleted below 40 CD19^+^ cells/μL and no signs of disease activity. Then we extend treatment intervals up to 9 or 12 months. Among older patients (and those having IgG in the lower range) we extend treatment intervals even further up to 15-24 months without taking into account reappearance of CD19 cells.

### Flow cytometric analysis

During the years 2012 until 2023, differential counts and classification of leukocytes were performed on a Navios (Beckman Coulter) with an IVD tetraCHROME kit (Beckman Coulter). From the year 2023, they were performed on a FACSLyric (BD Biosciences) with an IVDR BD 6 color Multitest with Trucount™ beads (BD Biosciences).

Patient’s differential counts and classification/differentiation assessments were performed with tetraCHROME CD45-FITC/CD4-PE/CD8-ECD/CD3-PC5 (Beckman Coulter), tetraCHROME CD45-FITC/CD56-PE/CD19-ECD/CD3-PC5 (Beckman Coulter) and Flow-Count Fluorospheres (Beckman Coulter) and was analyzed on a Navios flow cytometer (Beckman Coulter) up until the year 2023. After January 2023 differential counts were performed with a BD Multitest™ 6-color TBNK Reagent and BD Trucount™ (CD3-FITC, CD16-PE, CD56-PE, CD45-PerCP-Cy5.5, CD4-PE-Cy7, CD19-APC, CD8-APC-Cy7) and Trucount Absolute Counting Tubes and analyzed on a FACSLyric instrument (BD Bioscieneces). In brief, 20µl of BD multitest antibody cocktail is pipetted into BD Trucount tubes. 50µl of well mixed, anticoagulated whole blood is pipetted into the tube and incubated for 20 minutes in the dark and RT. 450µl of BD FACS lysing solution is added to tube and incubated for 20 minutes in the dark and RT. Samples are then analyzed on FACSLyric instrument in the FACSsuite clinical environment.

When samples were analyzed on Navios (Beckman Coulter), the protocol was similar, except that the counting beads were added to the vials as a final step, while the Trucount™ tubes already have beads. Gating methods for differential counts and classification/differentiation are shown in **Supplementary Figure 4**.

For the phenotypical analysis from 2012-2023 50 µl of whole blood was collected in two tubes and washed in 1ml of PBS, 300g 5 min, and stained for 30 min on ice, followed by a 10 min, RT, rbc lysis (BD biosciences) and washed twice (300g 5 min). The cells were resuspended in 350ul PBS and run on the flow cytometer. Cells were stained with the following antibodies tube 1: CD45RA-FITC, CD197-PE, CD45-ECD, CD4-PC5, CD3-PC7, CD38-APC, CD8-APC Alexa Fluor 750, HLA-DR-PB. Tube 2: CD16-FITC, IgD-PE, CD20-PC5, CD27-PC7, CD14-APC Alexa Fluor 750, CD19-PB. All antibodies from Beckman Coulter except CD16-FITC from Agilent Technologies and analyzed on Navios. After 2023 the phenotypical assay was run on a FACSLyric with the following antibodies purchased from BD Biosciences CD45-APC-H7, CD3-BV786, CD3-BV786, CD4-BV711, CD8-V500, CD19-FITC, CD56-PE, CD45RA-PE-Cy7, CD197-APC, HLA-DR-R718, CD38-PerCP-Cy5.5, CD20-V450, CD27-BV605. Gating strategy for the phenotypical analysis are shown in **Supplementary Figure 5**.

### Statistical analysis

The statistical tests Mann-Whitney, 2-way ANOVA corrected with Šídák’s multiple comparisons test, Kruskal-Wallis test corrected with Dunn’s multiple comparisons test, and Wilcoxon test were performed in GraphPad Prism 9.5.1.

## Results

**Figure 1:**
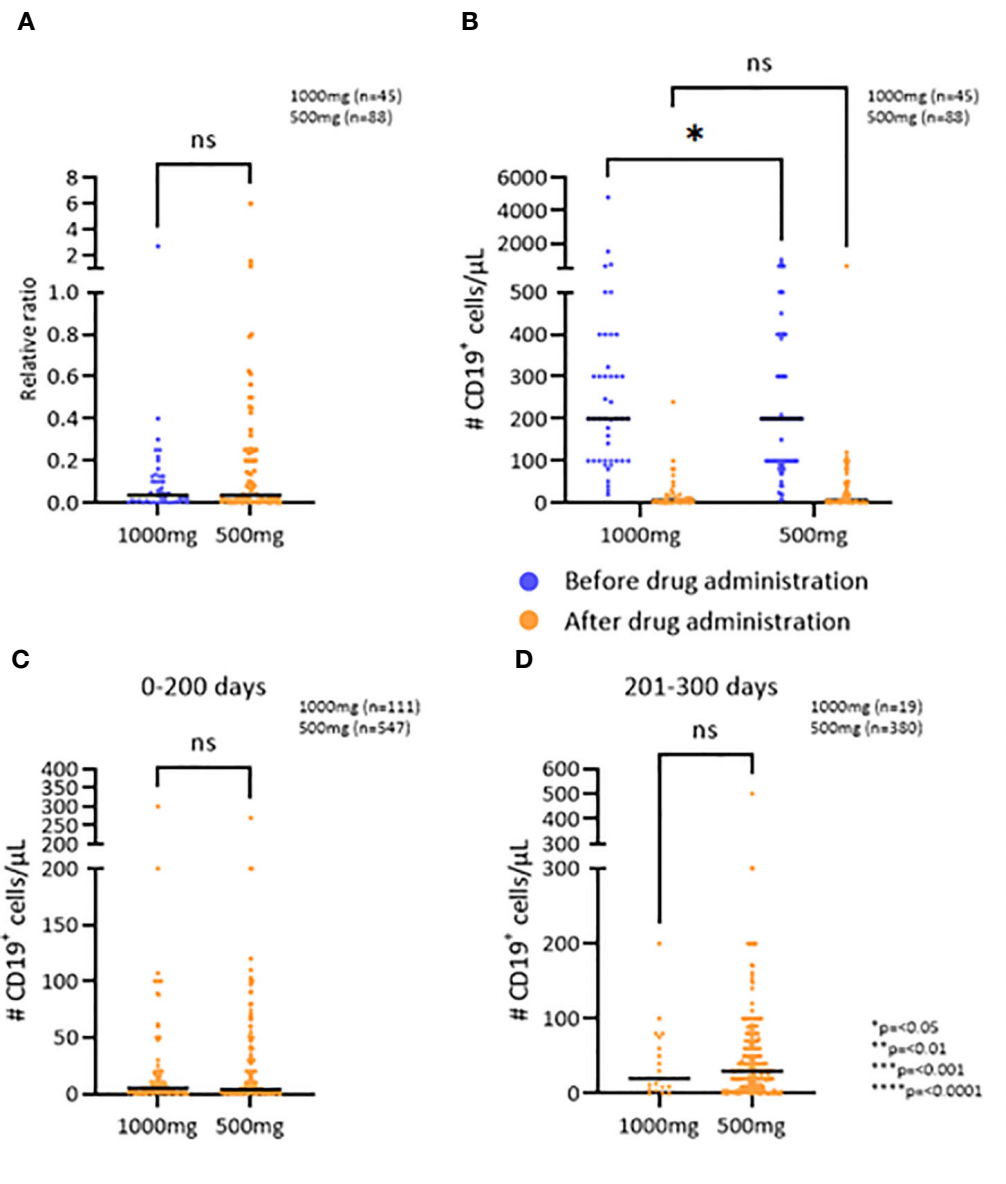
The effect of RTX on the B cell population. **(A)** The relative change in number of CD19^+^ cells/μL after the first RTX administration comparing two doses. **(B)** CD19^+^ cell depletion after the first administration of RTX comparing two doses. **(C, D)** Number of CD19^+^ cells/μL 0-200 and 201-300 days after the administration of RTX, respectively. *p=<0.05, **p=<0.01, ***p=<0.001, ****p=<0.0001. ns, not significant.

No difference was detected in the relative change of B cells (CD19^+^), when comparing the patient groups receiving an initial dose of either 500mg or 1000mg of RTX (p=0,2209) (**Figure 1A**), even though patients receiving 1000mg of RTX had a higher number of B cells before treatment (**Figure 1B**) (p=0,0341). There was no difference in RTX administration intervals between the two groups. B cell count was performed at an average of 6 months after RTX administrations for both groups, 500mg and 1000mg, respectively (**Supplementary Figure 1E**).

To examine if the observed effect of RTX on the persistence of B cell depletion was dependent on treatment dose or time since last administration, the two patient groups were further divided into three groups based on the time since the last RTX administration: 0-200 days, 201-300 days, and 301 or more days (**Figures 1C, D**; **Supplementary Figure 1F**). The patients who received 1000mg of RTX had an average of 5 CD19^+^ cells/μL in the first 200 days after administration and those who received 500mg had an average of 4 CD19^+^ cells/μL (p=0,7287) (**Figure 1C**). 200-300 days after RTX administration the patients who received 1000mg had an average of 20 CD19^+^ cells/μL and those who received 500mg had an average of 30 CD19^+^ cells/μL (p=0,6007). The average number of CD19^+^ cells more than 300 days after RTX administration was 40 CD19^+^ cells/μL, regardless of whether the patient received 1000mg or 500mg (p=0,9182). Thus, no difference was detected in the persistence of the B cell depletion when comparing the two initial doses.

The efficacy of RTX on B cell depletion was comparable between genders and between different age groups [16-29 yr, 30-59 yr and 60-84 yr (**Supplementary Figure 1B**)]. In addition, the long-term B cell depletion counts were not associated with age or gender (**Supplementary Figure 1A**). No difference in efficacy was detected between different RTX biosimilars (**Supplementary Figure 1H**).

**Figure 2:**
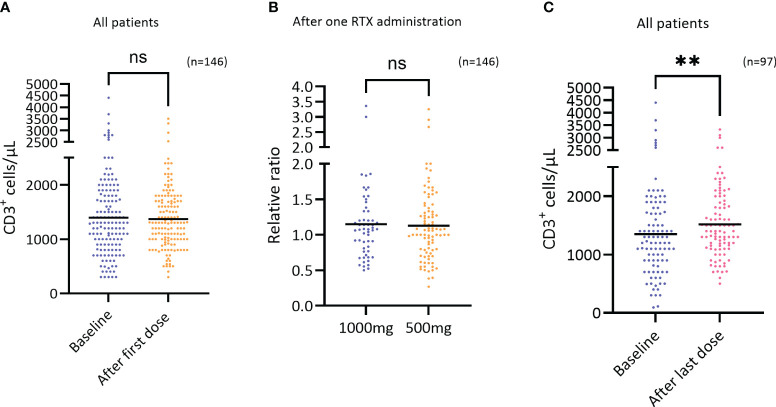
Short term effect of RTX on CD3^+^ cells. **(A)** The number of T cells (CD3^+^ cells/μL) at baseline and after the first RTX administration regardless of dosage (n=146), difference not significant. Wilcoxon test. **(B)** The relative change in the number of T cells (CD3^+^ cells/μL) after the first RTX administration comparing the two doses 1000mg (n=56) and 500mg (n=90), difference not significant. Mann-Whitney test. **(C)** The number of T cells (CD3^+^ cells/μL) at baseline and after the last RTX administration (3-8 RTX administrations) regardless of dosage (n=97), p=0.0084, Wilcoxon test. **p=<0.01. ns, not significant.

Upon evaluation of the T cell population, the number and percentages of T cells (CD3) were assessed at baseline and after one RTX administration. The results demonstrate that RTX treatment had no short-term effect upon these parameters (**Figures 2A, B**). However, by assessing the number of T cells at baseline and after the last RTX administration, ranging from 3-8 administrations and 1,5-8 years of treatment, the results demonstrate a median increase in the number of T cells of 167 cells/μL, or from 1351 to 1518 cells/μL (p=0.0084) (**Figure 2C**).

**Figure 3:**
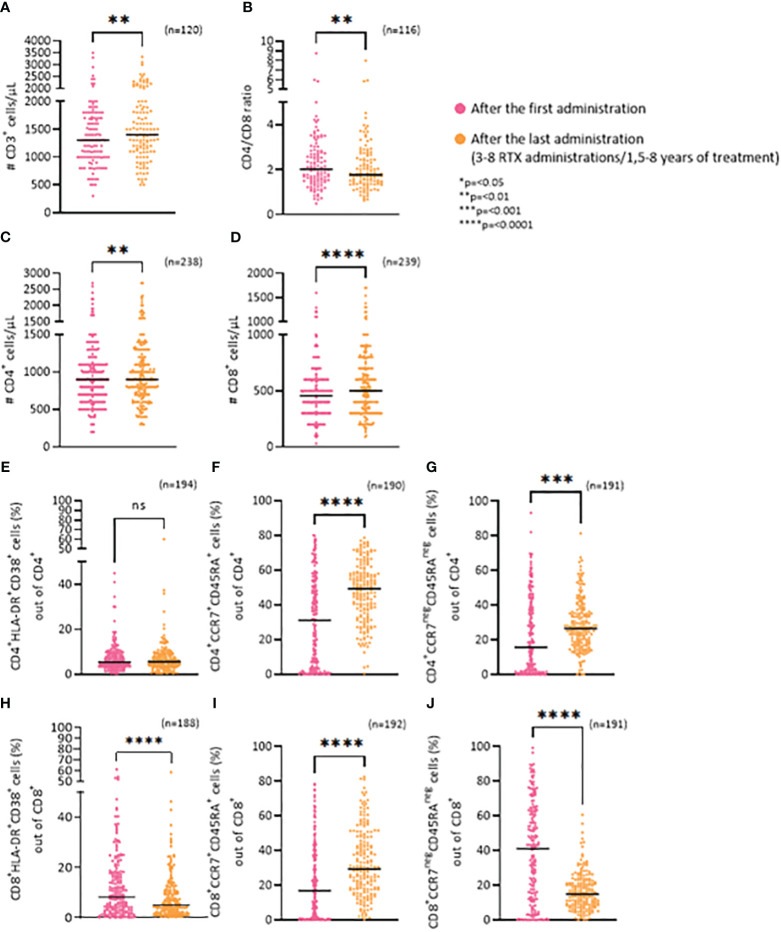
Long-term effects of RTX on T cells. **(A)** Frequency of CD3^+^ cells/μL. **(B)** CD4/CD8 ratio. **(C)** Frequency of CD4^+^ cells/μL. **(D)** Frequency of CD8^+^ cells/μL. **(E)** Percentage of CD4^+^/HLA-DR^+^/CD38^+^ (activated) cells. **(F)** Percentage of CD4^+^/CCR7^+^/CD45RA^+^ (näive) cells. **(G)** Percentage of CD4^+^/CCR7^neg^/CD45RA^neg^ (effector) cells. **(H)** Percentage of CD8^+^/HLA-DR^+^/CD38^+^ (activated) cells. **(I)** Percentage of CD8^+^/CCR7^+^/CD45RA^+^ (näive) cells. **(J)** Percentage of CD8^+^/CCR7^neg^/CD45RA^neg^ (effector) cells. *p=<0.05, **p=<0.01, ***p=<0.001, ****p=<0.0001. ns, not significant.

Assessing the number of T cells after the first and last RTX administration the results show that long-term use of RTX leads to a 9.5% increase of T cells numbers (p=0,0015) (**Figure 3A**), that is largely driven by CD8^+^ T cells also reflected by a significant decrease in the CD4/CD8 ratio (**Figure 3B**; p=0,004).

The average change in the number of CD4^+^ T cells was from 932 cells/μL to 977,9 cells/μL (p=0,0028) while it was 472,2 cells/μL to 537,95 cells/μL (p=<0,0001) for the CD8^+^ T cells (**Figures 3C, D**).

To further examine the CD8^+^ and CD4^+^ subsets, we analyzed the ratio of activated cells (HLA-DR^+^CD38^+^), näive cells (CCR7^+^CD45RA^+^) and effector cells (CCR7^neg^CD45RA^neg^). There was no change in the percentage of activated CD4^+^ cells (CD4^+^HLA-DR/CD38^+^), between the first and last RTX administration (**Figure 3E**). An increase was seen for the ratio of näive CD4^+^ cells (CD4^+^CCR7^+^CD45RA^+^) (p=<0,0001) (**Figure 3F**) and effector CD4^+^ cells (CD4^+^CCR7^neg^CD45RA^neg^) (p=0,0002) (**Figure 3G**). In contrast, RTX treatment led to a significant decrease in the percentage of both activated CD8^+^ cells (**Figure 3H**: CD8^+^HLA-DR^+^CD38^+^; p=<0,0001), and effector CD8^+^ cells (**Figure 3J**: CD8^+^CCR7^neg^CD45RA^neg^;p=<0,0001) between the first and last RTX administration. Whereas an increase in näive CD8^+^ cells (CD8^+^CCR7^+^CD45RA^+^) (p=<0,0001) (**Figure 3I**) was noted.

## Discussion

Taken together, these results demonstrate that a dose of 500mg of RTX is sufficient for B cell depletion in MS patients, as the results show that a dose of 500mg has similar impact as 1000mg on B cell depletion, a finding supported by others (2, 6). The persistence of B cell depletion does not seem to correlate with the dosage within the initial 200 days post-drug administration. However, research indicates that patients with higher B cell counts at baseline are less likely to attain complete B cell depletion, underscoring the necessity for personalized anti-CD20 treatment approaches (7). Further studies are needed focusing upon personalized dosing and time scheduling regimen that would, hopefully, eventually lead to enhanced therapeutic, and safety efficacy as well as improving its cost-effective outcome measures.

This study demonstrates that long-term use of RTX results in an increase of T cell numbers, most notably observed in the number of CD8^+^ T cell subset, leading to a significant decrease in the CD4/CD8 ratio. Interestingly, during long-term RTX treatment, a significant decrease in the percentage of both activated CD8^+^ cells and effector CD8^+^ cells was detected which seems to correlate with a drastic increase in the percentage of näive CD8^+^ cells. However, there is an increase in percentage of both näive CD4^+^ cells and effector CD4^+^ cells. Conversely, studies have shown that treatment using Ocrelizumab (OCR), another anti-CD20 antibody, has a depleting effect on the T cell populations, especially the CD8^+^ T cells (8–10). It is worth considering that the effects may vary between OCR and RTX treatment, OCR is a humanized antibody, while RTX is a chimeric monoclonal antibody. This calls into question whether the increase seen in T cell population after long-term use of RTX is due to the immunogenicity of the drug itself. Yahayzadeh et al. have previously shown a decrease in T follicular helper cells (Tfh) in MS patients after two doses of RTX (11). By analyzing the absolute numbers of the lymphocytes, we report an influx of CD4^+^ T cells, mostly with a naïve phenotype. Thus, a decrease in the percentages of Tfh cells will occur if there is an increase in the absolute numbers of naïve CD4^+^ T cells.

The variability in treatment duration (1,5-8 years) and the number of RTX administrations (3-8 administrations) in this retrospective study served as a strategy to maximize the depth of our data analysis. We aimed to capture a comprehensive representation of the long-term effects of RTX on the T cell population. This approach allowed us to expand our data significantly. In a controlled clinical trial setting, a fixed treatment duration and predetermined number of administrations can offer distinct advantages of further assessment of the long-term effects of RTX on the T cell population.

Patients on B cell depleting therapies for autoimmune diseases have been shown to have an altered immune response to vaccination, for example a lowered percentage of granzyme B^+^ CD8^+^ T cells in response to stimulation with the vaccine as compared to healthy controls (12). This may be due to the altered subgroups of CD8^+^ T cells in patients shown here, more naïve and less effector and activated. However, activated CD8^+^ T cells in patients receiving RTX treatment show similar responses to vaccination as in healthy controls (13).

In summary, long-term use of RTX is efficacious in suppressing the B cell population. However, our study demonstrates a long-lasting variable effect of RTX on selective T cell sub-populations. Whether these altered T cell subgroups play a significant role in the immunopathogenesis of MS and/or the therapeutic effectiveness of RTX, or is an immune response against the therapy itself, remains to be further investigated. In addition, these findings might help us enhance our understanding of the homeostatic milieu of within the lymphoid system during its repopulation process following B cell driven depletion protocol.

The finding that there is no difference in the efficacy of RTX on B cell depletion between 500mg or 1000mg suggests that 500mg is a sufficient dose and 1000mg could be avoided. Further studies are needed to assess whether doses below 500mg could suffice. The current study shows that the patients’ sex or age has no effect on the efficacy of RTX, but the optimal dosage in reference to the patients’ weight has not yet been established. Increased body weight has shown to contribute to a faster clearing of rituximab (14). A personalized dosing regimen of RTX for each patient, considering the weight of the patient, could be beneficial in terms of reducing the risk of side effects such as risk of infections, change in the T cell populations and infusion reactions as well as lowering the cost of treatment.

## Data availability statement

The original contributions presented in the study are included in the article/**Supplementary Material**. Further inquiries can be directed to the corresponding author.

## Ethics statement

The studies involving humans were approved by Landspitali – The National University Hospital Scientific Research Committee (42/2023). The studies were conducted in accordance with the local legislation and institutional requirements. Written informed consent for participation was not required from the participants or the participants’ legal guardians/next of kin because the information obtained in the study was flow cytometric data that was part of routine investigation in the treatment of patients visiting the neurological ward. The information was obtained from the journals of the patients. 

## Author contributions

GB: Writing – original draft, Methodology, Investigation, Formal analysis. HS: Writing – original draft, Supervision, Methodology, Investigation. SM: Writing – original draft, Supervision, Conceptualization. BE: Writing – review & editing, Supervision, Project administration, Methodology. ÓS: Writing – review & editing, Resources. HH: Writing – review & editing, Resources. SS: Writing – review & editing, Resources. BL: Writing – review & editing, Resources. SB: Writing – review & editing, Writing – original draft, Visualization, Validation, Supervision, Software, Resources, Project administration, Methodology, Investigation, Funding acquisition, Formal analysis, Data curation, Conceptualization.
