# Supplementary material for: Long-term use of rituximab increases T cell count in MS patients
Source: Front Immunol. 2024 Jul 17;15:1412668. doi: 10.3389/fimmu.2024.1412668 (PMC11288840; doi:10.3389/fimmu.2024.1412668)
Supplement: Supplementary file 1 [file DataSheet_1.docx]

Supplemental data


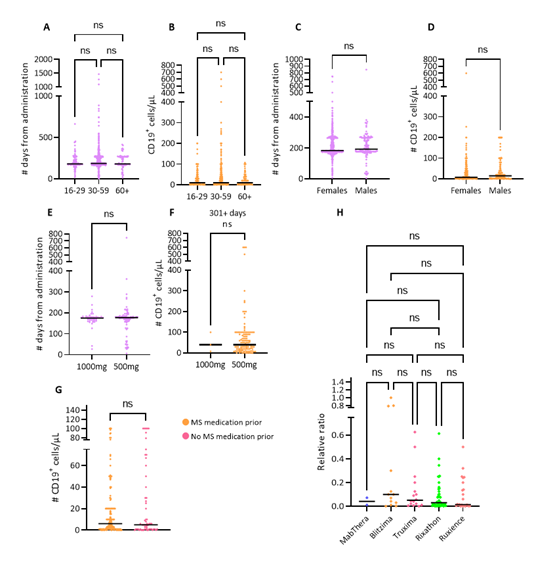


***Supplemental figure 1.*** *Supplemental data regarding the patients’ B cell population and intervals.* ***A)*** *The number of days between RTX administration and differential count results comparing the three age groups: 16-29 years old (n=187), 30-59 years old (n=1127) and over 60 years old (n=86), difference not significant.* *Kruskal-Wallis test.* ***B)*** *The number of B cells (CD19^+^ cells/μL) comparing the three age groups: 16-29 years old (n=187), 30-59 years old (n=1127) and over 60 years old (n=86), difference not significant.* *Kruskal-Wallis test.* ***C)*** *The number of days between RTX administration and differential count results comparing the sexes males (n=173) and females (n=441), difference not significant.* *Mann-Whitney test.* ***D)*** *The number of B cells (CD19^+^ cells/μL) comparing the sexes males (n=173) and females (n=441), difference not significant.* *Mann-Whitney test.* ***E)*** *The number of days between RTX administration and differential count results comparing the two doses 1000mg (n=45) and 500mg (n=88), difference not significant.* *Mann-Whitney test.* ***F)*** *The number of B cells (CD19^+^ cells/μL) more than 300 days after the last RTX administration comparing two doses 1000mg (n=3) and 500mg (n=127), difference not significant.* *Mann-Whitney test.* ***G)*** *The number of B cells (CD19^+^ cells/μL) after RTX administration comparing patients that had been treated for MS prior RTX treatment (n=206) and patients that had not been treated for MS prior RTX (n=61), difference not significant. Mann-Whitney test.* ***H)*** *The relative change in the number of B cells (CD19^+^ cells/μL) after the first RTX administration comparing different RTX brands: MabThera (n=2), Blitzima (n=13), Truxima (n=16), Rixathon (n=47), Ruxience (n=24), difference not significant. Kruskal-Wallis test.*


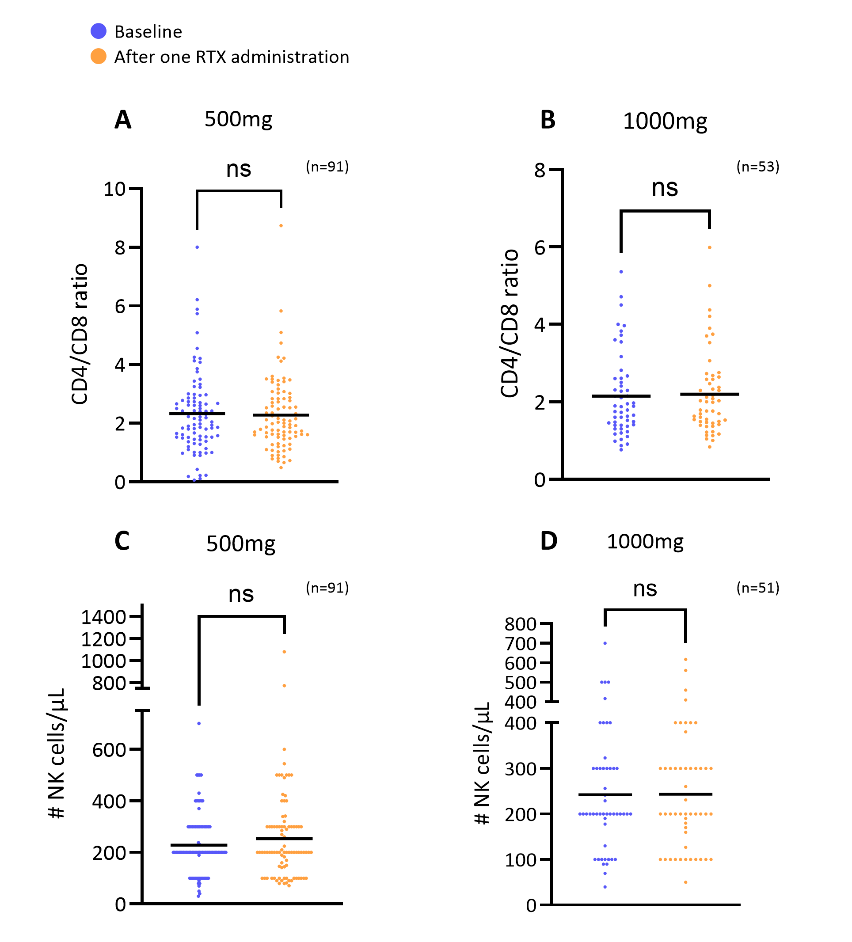


***Supplemental figure 2.*** *Short term effects of RTX on the CD4/CD8 ratio and the NK cell population.* ***A)*** *CD4/CD8 ratio before and after the first RTX administration of 500mg (n=91), difference not significant. Wilcoxon test.* ***B)*** *CD4/CD8 ratio before and after the first RTX administration of 1000mg (n=53), difference not significant. Wilcoxon test.* ***C)*** *The number of NK cells before and after the first administration of RTX og 500mg (n=91), difference not significant. Wilcoxon test.* ***D)*** *The number of NK cells before and after the first administration of RTX og 1000mg (n=51), difference not significant. Wilcoxon test.*


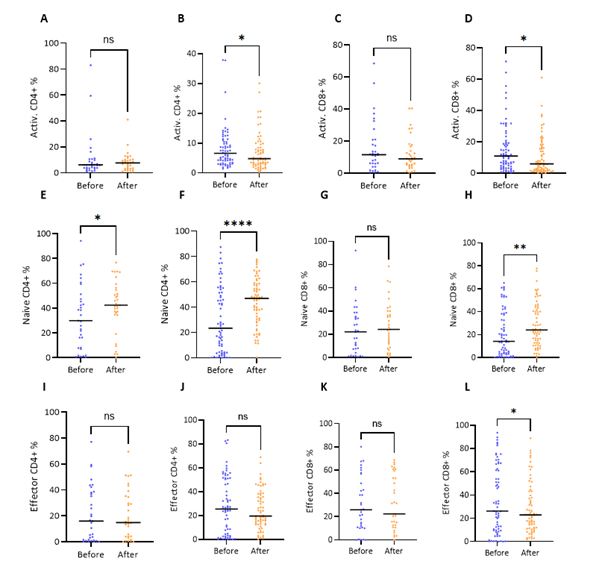


***Supplemental figure 3.*** *Short term effects of RTX on the T cell subsets.* ***A)*** *The change in the CD4^+^ percentage of activated CD4 T cells (CD4^+^HLA-DR/CD38^+^) before and after the first RTX administration of 1000mg (n=33), difference not significant. Wilcoxon test.* ***B)*** *The change in the CD4^+^ percentage of activated CD4 T cells (CD4^+^HLA-DR/CD38^+^) before and after the first RTX administration of 500mg (n=69), difference not significant. Wilcoxon test.* ***C)*** *The change in the CD8^+^ percentage of activated CD8 T cells (CD8^+^HLA-DR/CD38^+^) before and after the first RTX administration of 1000mg (n=34), difference not significant. Wilcoxon test.* ***D)*** *The change in the CD8^+^ percentage of activated CD8 T cells (CD8^+^HLA-DR/CD38^+^) before and after the first RTX administration of 500mg (n=71), difference not significant. Wilcoxon test.* ***E)*** *The change in the CD4^+^ percentage of näive CD4 T cells (CD4^+^CCR7^+^CD45RA^+^) before and after the first RTX administration of 1000mg (n=33), p=0.0305. Wilcoxon test.* ***F)*** *The change in the CD4^+^ percentage of näive CD4 T cells (CD4^+^CCR7^+^CD45RA^+^) before and after the first RTX administration of 500mg (n=67), p=<0.0001. Wilcoxon test.****G)*** *The change in the CD8^+^ percentage of näive CD8 T cells (CD8^+^CCR7^+^CD45RA^+^) before and after the first RTX administration of 1000mg (n=33), difference not significant. Wilcoxon test.* ***H)*** *The change in the CD8^+^ percentage of näive CD8 T cells (CD8^+^CCR7^+^CD45RA^+^) before and after the first RTX administration of 500mg (n=67), p=0.0016. Wilcoxon test.* ***I)****The change in the CD4^+^ percentage of effector CD4 T cells (CD4^+^CCR7^neg^CD45RA^neg^) before and after the first RTX administration of 1000mg (n=33), difference not significant. Wilcoxon test.* ***J)*** *The change in the CD4^+^ percentage of effector CD4 T cells (CD4^+^CCR7^neg^CD45RA^neg^) before and after the first RTX administration of 500mg (n=67), difference not significant. Wilcoxon test.* ***K)*** *The change in the CD8^+^ percentage of effector CD8 T cells (CD8^+^CCR7^neg^CD45RA^neg^) before and after the first RTX administration of 1000mg (n=33), difference not significant. Wilcoxon test.* ***L)*** *The change in the CD8^+^ percentage of effector CD8 T cells (CD8^+^CCR7^neg^CD45RA^neg^) before and after the first RTX administration of 500mg (n=68), p=0.0489. Wilcoxon test.*


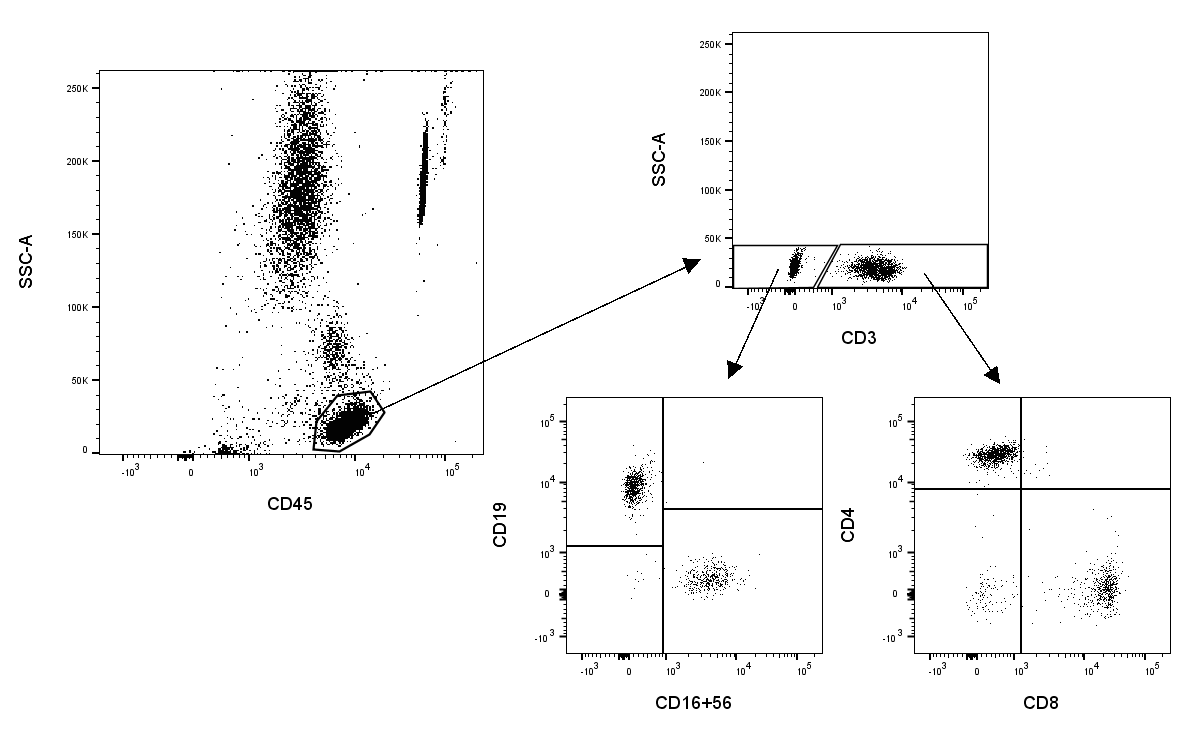


***Supplemental fig. 4. Gating methods for differential count*** ***and classification/differentiation assessments.*** *Differential counts were performed with a BD Multitest™ 6-color TBNK Reagent and BD Trucount™ (CD3-FITC, CD16-PE, CD56-PE, CD45-PerCP-Cy5.5, CD4-PE-Cy7, CD19-APC, CD8-APC-Cy7) and Trucount Absolute Counting Tubes and analysed on a FACSLyric instrument with the FACSuite clinical software (BD Bioscieneces).*


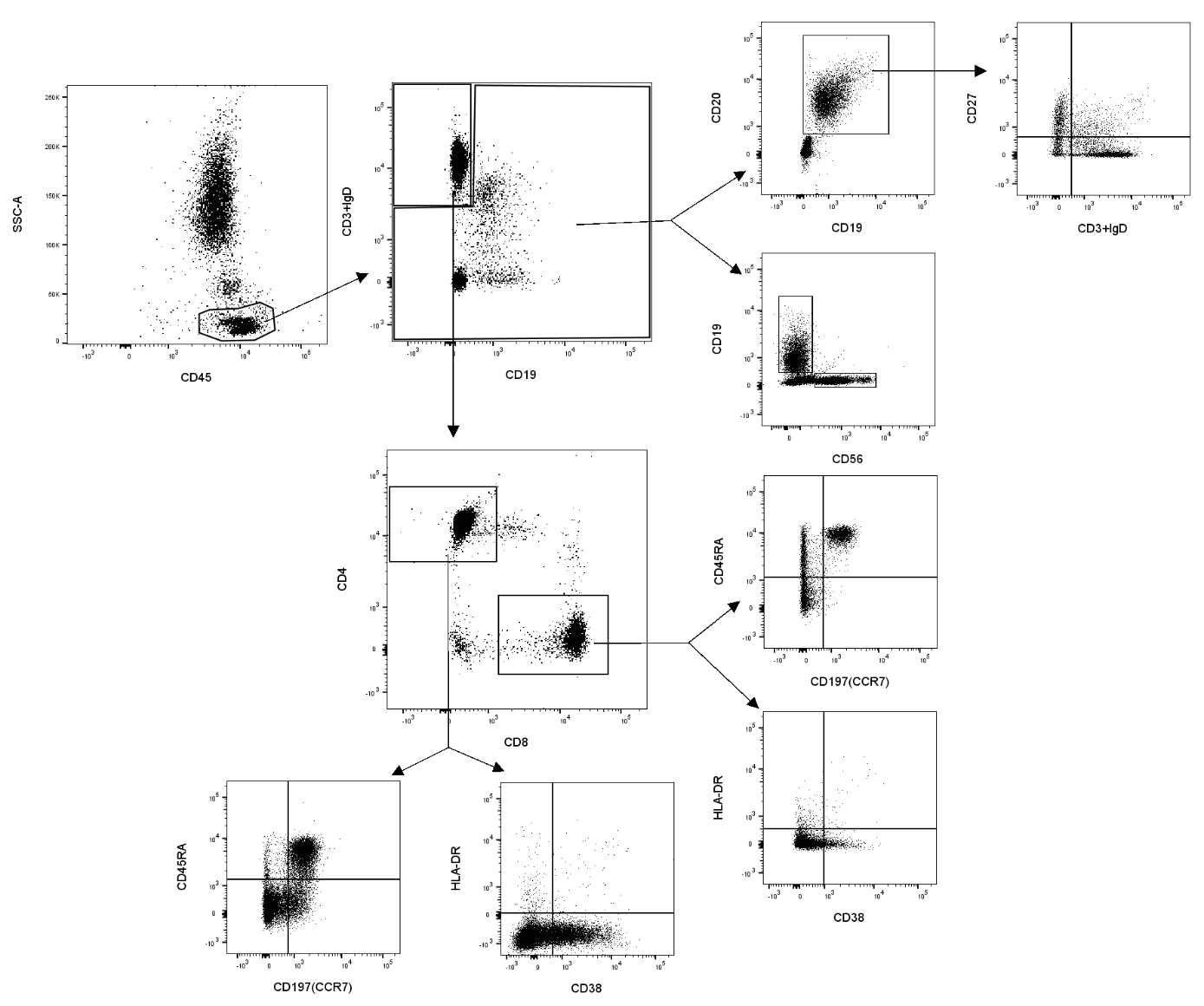


***Supplemental fig. 5. Gating strategy for the phenotypical assessments.*** *The phenotypical assay was run on a FACSLyric instrument with the FACSuite software. The following antibodies purchased from BD Biosciences CD45-APC-H7, CD3-BV786, CD3-BV786, CD4-BV711, CD8-V500, CD19-FITC, CD56-PE, CD45RA-PE-Cy7, CD197-APC, HLA-DR-R718, CD38-PerCP-Cy5.5, CD20-V450, CD27-BV605.*
